# Study on the transcriptional regulatory mechanism of the MyoD1 gene in Guanling bovine

**DOI:** 10.1039/c7ra11795g

**Published:** 2018-04-03

**Authors:** Di Zhou, Houqiang Xu, Wei Chen, Yuanyuan Wang, Ming Zhang, Tao Yang

**Affiliations:** Key Laboratory of Animal Genetics, Breeding and Reproduction in the Plateau Mountainous Region, Cell and Molecular Biology (PhD), Animal Department, Guizhou University Guiyang 550025 China dizhougz@163.com chenweigzu@163.com yywang0829@163.com 690816060@qq.com 1037901148@qq.com gzdxxhq@163.com; College of Life Science, Guizhou University Guiyang 550025 China

## Abstract

The MyoD1 gene plays a key role in regulating the myoblast differentiation process in the early stage of skeletal muscle development. To understand the functional elements of the promoter region and transcriptional regulation of the bovine MyoD1 gene, we cloned eight fragments from the sequence region of the MyoD1 gene promoter and inserted them into eukaryotic expression vectors for cotransfection with the mouse myoblast cell line C2C12 and Madin-Darby bovine kidney (MDBK) line. A variety of transcription factor binding sites in the longest 5′-flanking fragment from Guanling cattle MyoD1-P1 were predicted by using the online software TFSEARCH and ALGGEN PROMO as well as validated by the promoter-binding TF profiling assay II and yeast one-hybrid (Y1H) assay, including MyoD, VDR, MEF1, MEF2, SF1, and Myf6. Myf6 strongly activated the MyoD1 promoter, while MyoD1 was also capable of efficiently activating the expression of its own promoter. The transcription factors MEF2A, SF1, and VDR were further confirmed to be capable of binding to MyoD1 by Y1H system experiments. The effects of the Guanling cattle MyoD1 gene on the mRNA expression of the MEF2A, SF1, and VDR genes were determined by using a lentivirus-mediated overexpression technique, confirming that overexpression of the MyoD1 gene upregulated the mRNA expression of MEF2A as well as downregulated the expression of SF1 and VDR in the process of muscle myogenesis. Our study revealed the effects of transcription factors including MEF2A, SF1 and VDR on regulatory aspects of MyoD1, providing abundant information for transcriptional regulation of MyoD1 in muscle differentiation.

## Abstract 2

The MyoD1 gene plays a key role in regulating the myoblast differentiation process in the early stage of skeletal muscle development.

## Graphical abstract

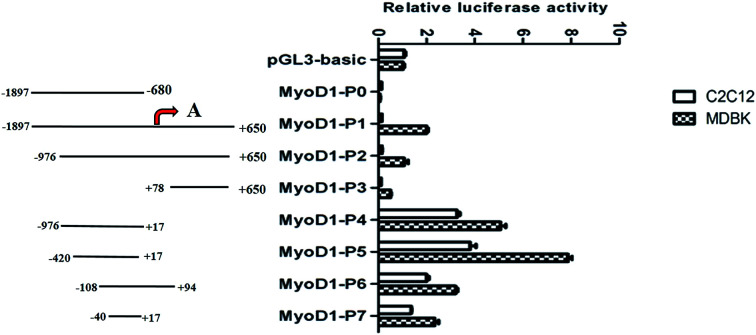

## 1. Introduction

Skeletal muscle is a complex bundle of multiple cells, called muscle fibers, with many sizes and various shapes. It consists of skeletal muscle tissue, connective tissue, nerve tissue, and blood or vascular tissue, and plays an important role in anatomical positioning, locomotion, preemption, mastication, and other dynamic events, including body metabolism regulation.^1^ Most skeletal muscles are attached to bones by bundles of collagen fibers.

Skeletal myogenesis is characterized by a series of discrete developmental events that regulate the establishment of stable stem cell lineages, differentiation into diverse myoblasts, and specialization of the contractile unit to slow-twitch (type I) and fast-twitch (type II).^2,3^ The study of skeletal muscle has centered on efforts to dissect the underlying molecular mechanisms that regulate the initial activation and subsequent modulation of the contractile protein gene set in the muscle development process, leading to understanding the regulation of muscle growth and quality in meat-producing animal species.^4–6^ Several transcriptional regulatory factors, including myoblast determination protein family members, are believed to act as terminal effectors of signaling cascades and to produce appropriate developmental stage-specific transcripts, regulating the development and growth of skeletal muscle.^7^

Skeletal myogenesis is regulated by the MyoD protein family, which includes four myogenic regulatory factors (MRFs)^8–11^ that belong to a family of muscle-specific basic helix-loop-helix (bHLH) transcription factors: myogenic differentiation (MyoD),^12^ myogenic factor 5 (Myf5),^13^ myogenin (MyoG),^14^ and myogenic regulatory factor 4 (MRF4, also known as Mrf6).^15^ Among the MRFs, the determination factor Myf5 is expressed before adoption of the myogenic fate. MyoD, induced by Myf5, regulates the differentiation potential of an activated myoblast by inducing cell cycle arrest, a prerequisite for myogenic initiation, and acts together with MyoG and myocyte enhancer factor 2 (MEF2) to drive differentiation.^16^ It is also involved in regulating the growth, development, and repair of skeletal muscle at the embryonic stage to maintain the relative stability of the individual skeletal muscle.^17,18^ MyoG expression happens later and is crucial for terminal differentiation, while Mrf4 promotes both fate determination and differentiation.^19,20^ MyoD and Myf5 play a leading role in the differentiation of stem cells into myoblasts, coordinating the formation of muscle cells.^10^ Obviously, these transcription factors do not act alone but exist as part of a complex network of mediators that control every stage of myogenesis.^21–24^ The expression of MRF genes displays subtle correlativity in myogenesis and is dependent on MyoD, showing synergistic effects among members of the MyoD gene family.^21^ Skeletal muscle differentiation takes place when MRF genes of the MyoD family are activated in muscle progenitors, and this genetic program is operative in both the trunk and head regions.^25^ Among the MRF genes, the myogenic differentiation 1 (MyoD1) gene, first cloned in 1987,^12^ encodes a nuclear protein that belongs to the bHLH family of transcription factors and the myogenic factor subfamily. As a well-known transcription factor involved in controlling myogenic processes, MyoD1 is widely used as a gene model for the regulation of myogenesis. Promoters are important regulatory elements that initiate transcription and regulate the intensity and specificity of transcriptional genes.^26,27^ Previous studies have revealed that MyoD1 can promote transcriptional activation to regulate the expression of muscle-specific genes, including MyoG, creatine kinase, myosin, and desmin, by binding to their promoter regions.^28,29^ Despite significant progress in understanding the regulatory mechanisms of MyoD-mediated myogenesis at the transcriptional level in mice, chickens, and pigs,^22,23,30^ the roles of the core MyoD1 gene promoter-regulatory regions in the regulation of muscle differentiation remain elusive in cattle.^31^

Skeletal muscle from domestic animals including cattle is a major source of high-quality protein in the human diet. Recent studies have investigated utilization of MyoD in animal breeding for meat improvement.^32–34^ Qiu *et al.* (2010) have reported the important role of MyoD in bovine muscle development.^35^ In addition, Zhang *et al.* (2015) have reported that the Guanling bovine MyoD1 gene promoter region contains binding sites for the transcription factors MyoD, transcription factor IID (TFIID), Pax3, myocyte enhancer factor 1 (MEF1), MEF2, and vitamin D receptor (VDR);^36^ however, the effects of these transcription factors on the MyoD1 gene promoter are not clear. This experiment was designed to investigate the effects of the core MyoD1 gene promoter-regulatory regions on the regulation of bovine muscle differentiation by binding to transcriptional factors. In the present study, we successfully cloned the core coding region of the Guanling bovine MyoD1 gene promoter and overexpressed it in the mouse myoblast cell line C2C12 and Madin-Darby bovine kidney (MDBK) line to understand the transcriptional regulation mechanisms of the MyoD1 gene promoter-binding transcriptional factors, including myocyte enhancer factor 2A (MEF2A), steroidogenic factor 1 (SF1), and VDR, in skeletal myogenesis. Our study revealed the effects of transcription factors including MEF2A, SF1 and VDR on regulatory of MyoD1, providing abundant information for transcriptional regulation of MyoD1 in muscle differentiation.

## 2. Results

### 2.1. A region of the Guanling bovine MyoD1 gene contains putative muscle-specific promoter-regulatory elements

**Fig. 1:**
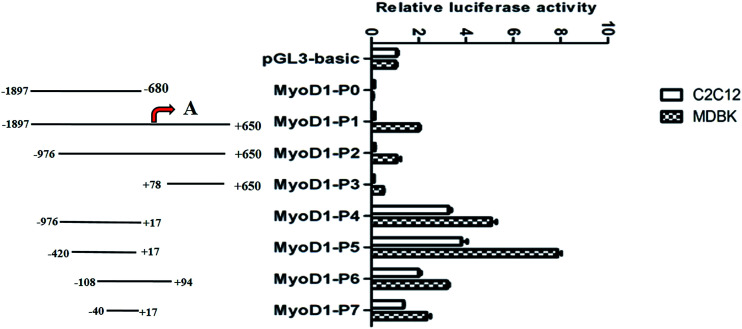
Measurement of the double fluorescence activity of the coding DNA sequence of the Guanling cattle MyoD1 gene promoter in the mouse myoblast cell lines C2C12 and MDBK. The left panel shows the corresponding region of the bovine MyoD1 gene promoter inserted into the pGL3-basic vector. The predicted transcription start site (position of the red arrow) is marked as +1. PGL3-basic was used as a negative control, while pRL-TK was used as the reference vector. The data are represented as the mean ± standard deviation of three replicates.

To begin to discover the muscle-specific regulation of MyoD1 gene transcription, we cloned a total of eight fragments of different lengths from a region of the MyoD1 gene promoter and inserted them into the pGL3-basic reporter plasmid. The fragments were as follows: −1897/−680 (MyoD1-P0), −1897/+650 (MyoD1-P1), −976/+650 (MyoD1-P2), +78/+650 (MyoD1-P3), −976/+17 (MyoD1-P4), −420/+17 (MyoD1-P5), −108/+94 (MyoD1-P6), and −40/+17 (MyoD1-P7), relative to the predicted transcription start site at +1 (Fig. 1). The constructs were transiently transfected into the mouse myoblast cell lines C2C12 and MDBK to estimate the levels of luciferase activity. In the C2C12 cell line, no luciferase activity was found in three constructs (MyoD1-P0, MyoD1-P1, and MyoD1-P3), while an increase of activity was detected in MyoD1-P4, the highest activity was identified in MyoD1-P5, and then a reduced activity was observed in MyoD1-P6, followed by MyoD1-P7. As shown in Fig. 1, a similar expression pattern of fluorescence activity was also observed in the MDBK cell line. Taken together, these data strongly indicate that the region of nucleotides −420 to +17 bp is the promoter-regulatory core region, which plays an important role in the transcriptional activity of the MyoD1 gene.

### 2.2. The region of the MyoD1 gene that mediates muscle-specific expression

A variety of transcription factor binding sites in the longest 5′-flanking fragment from Guanling cattle MyoD1-P1 were predicted by using the online software TFSEARCH (http://diyhpl.us/∼bryan/irc/protocol-online/protocol-cache/TFSEARCH.html) (Table 1) and ALGGEN PROMO (http://alggen.lsi.upc.es/) (Table 2). The Guanling cattle MyoD1 promoter contained a variety of myogenic transcription factor binding sites, including the MRF gene family (MyoD, MyoG, Myf5, and Myf6), MEF2 gene family (MEF2A, MEF2B, MEF2C, and MEF2D), SF1 gene, and VDR gene (Tables 1 and 2), among them, only MEF2, SF1 and VDR are predicted to bind to the functional region of the promoter (fragment −976 – +17).

**Table 1:** The transcription factor binding sites in the bovine MyoD1 gene promoter predicted by the online software TFSEARCH

Position (bp)	Transcription factor binding sites
1–200	SRY, CdxA, GATA-2, GAATA-1, AML-la, p300, Pbx-1, GATA-3, MEF2, Lyf-1
200–400	C/EBP, HSF2, IK-2, HSF1, CdxA, STATx
400–600	CdxA, STATx, HSF2, MEF2
600–800	HNF-3b, CdxA, AML-la, GATA-1, GATA-3, SRY
800–1000	CdxA, MEF2, AML-la, GATA-1, deltaE, EIK-1, USF, C/EBPb, C/EBPa
1001–1200	GATA-2, MEF2, C/EBP, GATA-1, SF1, HNF-3b, GATA-3, Sp1
1201–1400	AML-la, Nkx-2, AP-1, MEF2, CdxA, CP2
1401–1600	GATA-2, MEF2, CdxA, GATA-1, GATA-3, E2F, C/EBP
1601–1800	MEF2, Nkx-2, CP2, IK-2, NF-Kap, Sp1
1801–2000	CdxA, MEF2, NF-Kap, Sp1, MyoD, E2, GATA-2, GATA-3
2001–2200	GATA-1, GATA-2, GATA-3, Nkx-2
2201–2547	USF, N-Myc, Sp1, HSF2, P300, CdxA, SRY

**Table 2:** The transcription factor binding sites in the bovine MyoD1 gene promoter predicted by the online software ALGGEN PROMO

Position (bp)	Transcription factor binding sites
1–200	p300, GATA-2, YY1, HMG I(Y), STAT4, ELF-1, MEF2, GATA-1, STAT5A, AR, c-Ets-2, SRY, VDR
200–400	p300, GATA-2, STAT4, GATA-1, GATA-3, NF-X3, c-Jun, TFIIB, STAT5A, p53, TFIID, Pax-2
400–600	p300, YY1, STAT4, TFIIB, STAT5A, SRY, AP-3
600–800	p300, STAT4, c-Ets-1, GATA-1, NF-X3, TFIIB, STAT5A, SRY, TBP, TMF, MyoD, TFIID
800–1000	AP-3, Myf-3, MyoD, Pax-2, c-Myb, factor, SXR:RXR-alpha, RelA, NF-AT3, NF-AT2, NF-AT1, Pax-5
1001–1200	YY1, STAT4, GATA-1, STAT5A, VDR, E47, NFI/CTF, p53, AP-3, NF-1, Myf-3, MyoD, c-Myb
1201–1459	p300, WT1 I, LCR-F1, CUTL1, C/EBPalpha, C/EBPbeta, Elk-1
1401–1600	p300, WT1 I, PR B, PR A, SF1, GATA-2, Elk-1, R2, STAT5A, Pax-2, Pax-5
1601–1800	Sp3, Myf-3, MyoD, USF2b, TGIF, c-Fos, Pax-2, c-Myb, E2F-1, SXR:RXR-alpha
1801–2000	Nkx2-1, HNF-3beta, MEF2, GATA-1, GATA-3, TFIIB, PPAR-alpha:RXR-alpha, VDR, Myf-3, MyoD, TFIID
2001–2200	p300, GATA-2, YY1, MZF-1, GATA-1, VDR, E47, p53, Sp3, Pax-2, c-Myb, Sp1
2201–2400	p300, STAT4, STAT5A, VDR, p53, Sp3

**Fig. 2:**
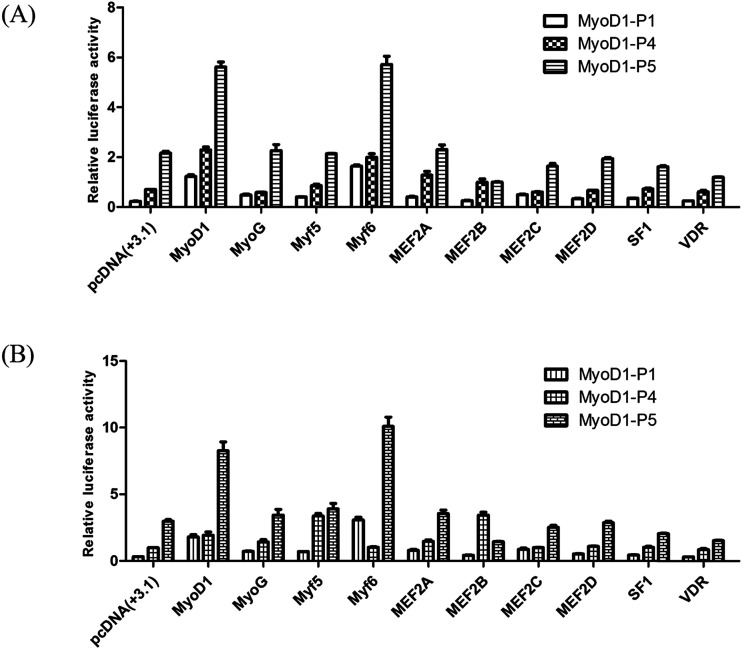
Effects of different transcription factors on the double fluorescence activity of the coding DNA sequence of the Guanling cattle MyoD1 gene promoter. (A) C2C12 cells. (B) MDBK cells. The mixed plasmid DNAs (200 ng of MyoD1-P(−) + 200 ng of transcription factors) were used to cotransfect the cell line. pGL3-basic was used as a negative control, while the pRL-TK vector was used as an internal reference vector. The data are represented as the mean ± standard deviation of three replicates.

To investigate if these transcription factors were involved in activation of the Guanling cattle MyoD1 promoter, we used the promoter-binding TF profiling assay to detect both direct- and indirect-TF binding sites to the DNA. MyoD1-P4 and MyoD1-P5 with higher luciferase activity were cotransfected with selectively responsive transcription factors into the mouse myoblast cell lines C2C12 and MDBK. The fluorescence activity showed that the MyoD1 and Myf6 genes could significantly increase the activity of the MyoD1 gene in the C2C12 and MDBK cell lines (Fig. 2). As shown in Fig. 2, Myf6 mediated the strongest activation of the MyoD1 promoter. Meanwhile, MyoD1 was able to activate its own promoter in this assay. The effects of the different transcription factors on the activity of MyoD1-P5 were higher than that of MyoD1-P4. The results strongly confirmed the transcriptional activity of different fragments of MyoD1 gene promoter above, the MyoD1 transcription start site was in region of the MyoD1-P5 sequence (nucleotides −420 to +17 bp) where transcription was initiated (Table 3).

**Table 3:** Sequences of the PCR primers used for amplification of the coding DNA sequence of the Guanling cattle MyoD1 gene promoter

Gene symbol	Forward primer (5′ to 3′)	Reverse primer (5′ to 3′)	Length (bp)	*T*_m_ (°C)
MyoD1-P0	ACCTCCCGACATCATACATT	GAAACCCAGCCGCATCTA	1217	58
MyoD1-P1	ACCTCCCGACATCATACATT	GGTTTGGGTTGCTAGACG	2547	60
MyoD1-P2	GTGGAGTTCCGCTTGTTG	GGTTTGGGTTGCTAGACG	1626	58
MyoD1-P3	GATATGGAGCTGCTGTCGC	AGCCGCTGGTTTGGGTTGC	728	59
MyoD1-P4	GTGGAGTTCCGCTTGTTG	CTCCCCACCCCTACTTTC	993	58
MyoD1-P5	CTCCCTGATTCGGTAGATC	CTCCCCACCCCTACTTTC	437	58
MyoD1-P6	CTCCCTGCTCTGTTCCTATT	AAACTTGCTGCTGTTCTGG	202	58
MyoD1-P7	TTAGGCTACTACGGGATAAA	CTCCCCACCCCTACTTTC	57	58

### 2.3. The transcription factors MEF2A, SF1, and VDR bind to the Guanling cattle MyoD1 gene promoter

#### 2.3.1 Proof from the promoter-binding TF profiling assay II

**Fig. 3:**
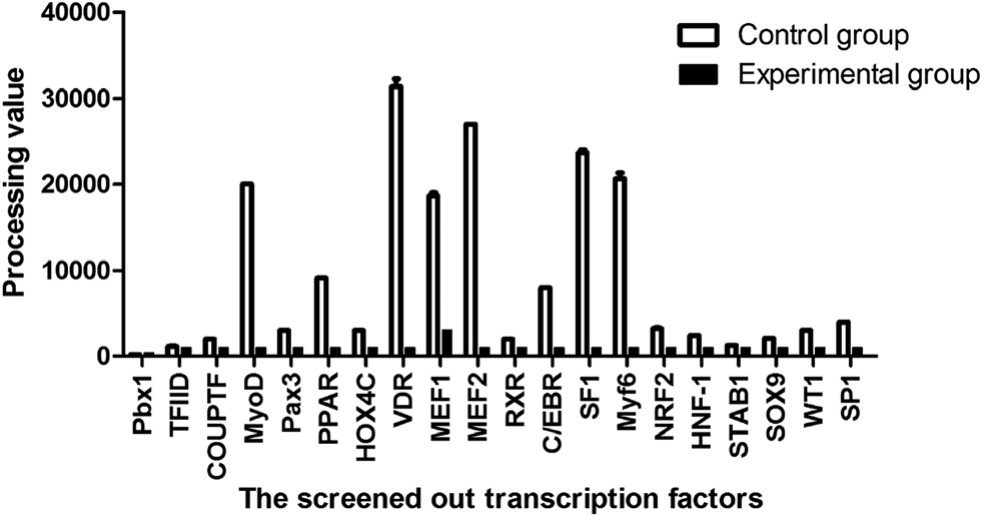
The screening results of the promoter-binding TF profiling assay II between the treatment and control groups. The processing of the statistically treated data was described in 4.5 Statistical analysis.

The experimental data were measured by a Multifunctional Microplate Reader (BioTek, Winooski, VT, USA) and statistically processed to represent the intensity of luminosity, meaning the amount of washed-off probe in the nuclear extracts after binding of each of the transcription factors to the corresponsive probe, indirectly indicating whether the promoter carries the transcription factor binding sites. As shown in Fig. 3, VDR showed the highest intensity of luminosity, followed by MEF2, SF1, Myf6 and MyoD. Taken together, the statistically treated data (Fig. 3) preliminarily showed that the promoter of the MyoD1 gene contains multiple putative transcription factor binding sites for MyoD, VDR, MEF1, MEF2, SF1, and Myf6 (Table 4).

**Table 4:** Sequences of the PCR primers used for amplification of transcription factors

Gene symbol	Forward primer (5′ to 3′)	Reverse primer (5′ to 3′)	Length (bp)	*T*_m_ (°C)
Myf5	ATGGACATGATGGACGGCTG	TCATAGCACATGATAGATG	786	58
Myf6	ATGATGATGGACCTTTTTGAAACTGGC	TTACTTCTCCACCACCTCCTCCACGCAG	745	58
MyoD1	ATGGAGCTGCTGTCGC	TCAGAGCACCTGGTAAAT	975	62
MyoG	ATGGAGCTGTATGAGACCTCT	TCAGTTTGGTATGGTTTCATCTGG	675	59
MEF2A	ATGGGGCGGAAGAAAATACAAATCA	TTAGGTCACCCACGCATCCATCCGC	1497	61
MEF2B	ATGCGAGATCGCCCTCATCATCTTC	CATCGCAGAGACAGTGGTACTGCTG	1107	60
MEF2C	ATGCAGACGATTCAGTAGGTCACAG	CTATCTATTGTAACATACATTTTGC	1425	62
MEF2D	ATGGGGAGGAAAAAGATTCAGATCC	TCACTTTAATGTCCAGGTGTCGAGT	1524	62
SF1	ATGGCGACCGGAGCGAACGCTACGC	CTAGTTCTGTGGTGGAGGCGGTGGG	1920	61
VDR	ATGGAGGCGACTGCGGCCAGCACTT	CTAGGAGATCTCGTTGCCAAACACCTC	1281	60

#### 2.3.2 Evidence from the yeast one-hybrid (Y1H) system

The bait vector pAbAi-MyoD1 was digested with BbsI to produce linearized products that were transformed into Y1H competent cells. The yeast transformants were screened on synthetic dextrose (SD)/-uracil (Ura) plates and further identified using colony polymerase chain reaction (PCR), confirming the successful transformation.

The fragments amplified from the MEF2A and VDR that are proved to be involved in myogenic mechanism,^36,47^ and SF1^56^ in the functional region of the promoter were recovered from the gel and ligated with the pGADT7 vector to construct the prey plasmids pGADT7-SF1, pGADT7-MEF2A, and pGADT7-VDR. PCR confirmed that the fragments of the MEF2A, SF1, and VDR genes were successfully inserted into the pGADT7 vector.

**Fig. 4:**
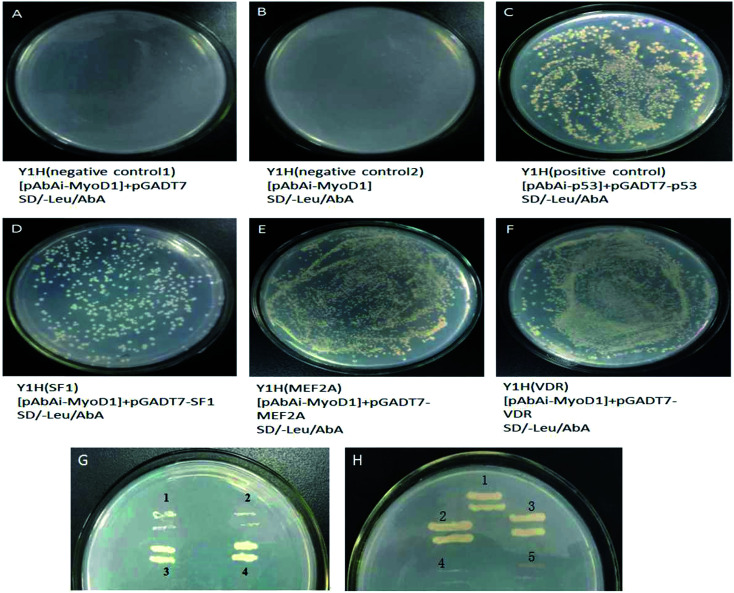
The colonies of Y1H bait strains transfected with prey vectors on SD/-Leu/AbA plates containing 200 ng mL^−1^ AbA. (A) Y1H (negative control 1) transfected with pAbAi-MyoD1 + pGADT7. (B) Y1H (negative control 2) transfected with pAbAi-MyoD1. (C) Y1H (positive control) transfected with pAbAi-p53 + PGADT7-53. (D–F) Y1H transfected with pAbAi-MyoD1 + pGADT7-SF1 (D), pAbAi-MyoD1 + pGADT7-MEF2A (E), and pAbAi-MyoD1 + pGADT7-VDR (F). (G) Streaking cultivations of negative controls (1 and 2), Y1H transfected with pAbAi-MyoD1 + pGADT7-SF1 (3), and a positive strain (4). (H) Streaking cultivations of positive control (1), Y1H transfected with pAbAi-MyoD1 + pGADT7-MEF2A (2), Y1H transfected with pAbAi-MyoD1 + pGADT7-VDR (3), and negative controls (4 and 5).

The prey plasmids (pGADT7-SF1, pGADT7-MEF2A, and pGADT7-VDR) and the pGADT7 empty vector were transfected into the bait strains. The transfected strains were inoculated on SD/-leucine (Leu)/Aureobasidin A (AbA) plates. As shown in Fig. 4, the colonies were observed to grow uniformly on the plates spread with strains transfected with pGADT7-SF1, pGADT7-MEF2A, and pGADT7-VDR. Positive transformants were confirmed by PCR, suggesting the successful transfection of the prey plasmids into the bait strains and activation of the transcription of the AbA resistance gene in bait strains by interactions between the prey protein and the bait gene. The bait strains transfected with the pGADT7 empty vector were unable to grow on SD/-Leu/AbA plates. No colonies were observed on the SD/-Leu/AbA plates incubated with bait strains, meaning no occurrence of self-activation of transcription of the AbA resistance gene. These results confirmed that the MEF2A, SF1, and VDR genes were the transcription factors binding to the MyoD1 gene promoter.

### 2.4. Overexpression of the MyoD1 gene upregulated the mRNA expression of MEF2A but downregulated SF1 and VDR expression

**Fig. 5:**
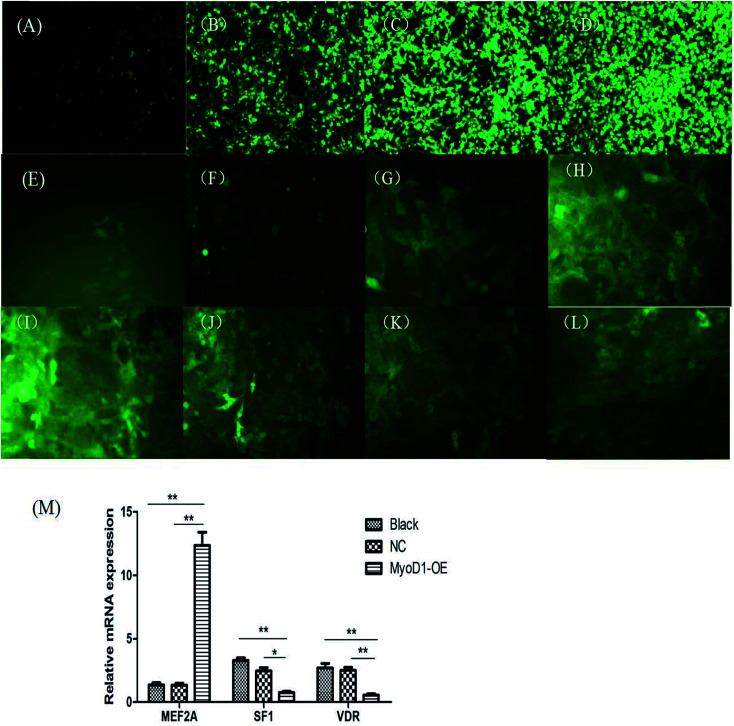
Overexpression of the lentiviral overexpression vector pCDH-CMV-MyoD1-EF1-GFP + Puro in *E. coli*. (A–D) The fluorescence pictures of 293T cells transfected with pCDH-CMV-MyoD1-EF1-GFP at 6 h before transfection (A) as well as 24 h (B), 48 h (C), and 72 h (D) after transfection. (E–H) The fluorescence pictures of MDBK cells transfected with pCDH-CMV-MyoD1-EF1-GFP at 6 h before transfection (E) as well as 24 h (F), 48 h (G), and 72 h (H) after transfection. (I–L) The stable cell lines of second (I), third (J), fourth (K), and fifth (L) generation cells overexpressing the MyoD1 gene. The screening concentration of puromycin was 1 μg mL^−1^. Magnification: 20×. (M) Effects of MEF2A, SF1, and VDR genes on mRNA expression of the overexpressed MyoD1 gene-stabled cell lines (*P < 0.05; **P < 0.01, ***P < 0.001).

The fragment amplified from the coding DNA sequence region of the MyoD1 gene was used to construct the lentiviral overexpression vector pCDH-CMV-MyoD1-EF1-GFP + Puro. As shown in Fig. 5, we generated MyoD1 gene-overexpressing stable cell lines. Overexpression of the MyoD1 gene significantly increased expression of the MEF2A gene but decreased expression of the SF1 and VDR genes. These results suggested that overexpression of the MyoD1 gene upregulated the mRNA expression of the MEF2A gene and downregulated the mRNA expression of the SF1 and VDR genes in the process of muscle myogenesis.

## 3. Discussion

This study provided evidence for direct activation of the bovine MyoD1 promoter by the muscle-specific transcription factor Myf6. We demonstrated that a small region of the MyoD1 gene surrounding the transcription start site directs muscle-specific expression. Among the 10 transcription factors evaluated in the present study, Myf6 strongly activated the MyoD1 promoter, while MyoD1 also was capable of efficiently activating the expression of its own promoter. Black *et al.* (1995) have reported that the Myf6 promoter is activated directly by other bHLH muscle-specific transcription factors, including MyoD1, but was unable to activate its own promoter in mice.^37^ In the present study, the Guanling cattle MyoD1 gene was proven to be directly activated by Myf6, and further experiments should be designed to study whether the MyoD1 gene can be indirectly activated by other transcription factors through indirect pathways. The MyoD gene family (MyoD1, Myf5, MyoG, and Myf6) has been shown to act as a key regulator that controls the expression of specific proteins in the proliferation and differentiation of muscle cells.^8–11,18,27^ Among them, MyoD1 plays an important role in muscle growth in the transcriptional regulation of muscle-specific genes,^38^ while Myf6 (MRF4) primarily functions in a downstream role in myogenesis, including myofiber formation^19,39^ and the maintenance of the muscle phenotype.^39^ The Myf6 and MyoD1 promoters are regulated quite differently, leading to opposite roles of MRF4 and MyoD in cell proliferation and myogenic differentiation,^40^ in which MyoD is a potential negative intercessor of MRF4 in regulating the cell cycle. Thus, while all of the myogenic bHLH factors are able to mediate myogenesis, it is becoming clear that they are regulated by different mechanisms.

We have reported that the Guanling cattle MyoD1 gene promoter contains multiple putative transcription factor binding sites, including MyoD, VDR, MEF1, MEF2, SF1, and Myf6. This finding is partially consistent with the results of Zhang *et al.* (2015, 2016),^36,41^ who have reported a number of transcription factor binding sites, including MyoD, MEF1, MEF2, Myf6 and VDR, in the Guanling bovine MyoD1 gene promoter. Zhou *et al.* (2016) reported that the action sites of transcription factors MyoD, Myf5 and Myf6 of Guanling cattle are not on core region of with *MyoD*1 gene promoter. In the present study, the key transcription factor binding sites VDR and MEF2A that are proved to be involved in myogenic mechanism,^36,47^ and SF1 in the functional region of the promoter have been shown to be capable of binding to the MyoD1 gene promoter by the Y1H assay. The MEF2 family, which is widespread in muscle cells, belongs to the MADS superfamily and includes four structurally similar members, MEF2A, MEF2B, MEF2C, and MEF2D, playing a role in the activation of muscle-specific gene transcription.^7,42,43^ The MEF2 family acts as a major regulator of myogenic gene expression to activate or enhance gene expression by directly binding to many muscle-specific gene promoters or enhancers. Studies on the MEF2 gene families have mostly concentrated on MEF2A.^44–47^ The expression of MEF2 factors can be activated by members of the myogenic bHLH family, including MyoD,^48^ while the MEF2 binding site is required for the expression of several myogenic bHLH factors.^49^ Chen *et al.* (2016) revealed the relation of MEF2A to myogenic response by downregulating expression and activity of the uncoupling protein 3 (UCP3) promoter in Guanling bovine.^47^ In the present study, mRNA expression of the MEF2A gene was upregulated by overexpression of the MyoD1 gene; however, MEF2A was unable to activate the MyoD1 gene promoter directly. It is proposed that MEF2 factors activate the MyoD1 gene promoter through an indirect pathway and that MEF2A and MyoD1 appear to function in a complex network by auto- and cross-activating the expression of themselves as well as each other.

The present study discovered that the mRNA expression of SF1 and VDR was downregulated by the MyoD1 gene in the process of muscle myogenesis. VDR, a member of the nuclear receptor family of transcription factors,^50^ is believed to activate intracellular signaling pathways in skeletal muscle^51^ and to mediate the nongenomic effects of the steroid hormone 1a,25-dihydroxyvitamin D3 (1,25(OH) 2D3) in chick myoblasts.^52^ The results of the present study confirmed that VDR is a MyoD1 gene promoter binding transcription factor, but unable to significant increase activation of MyoD1 gene (Fig. 2). The receptor expressed, as shown in Fig. 2, could be inactive in the absence of the ligand. It is likely, therefore, that VDR affects MyoD1 gene promoter activity by binding to other transcription factors as a complex. SF1 is a member of the nuclear receptor family of intracellular transcription factors and mediates regulation of the human P450scc gene by p300/CBP,^53^ which has been demonstrated to mediate the activity of several factors such as MyoD.^54,55^ Whereas both VDR and SF1 were shown to be downregulated by the myogenic MyoD1 gene in the present study, the question of whether SF1 and VDR found in the Guanling cattle MyoD1 gene promoter region can also directly or indirectly associate with the activation of transcriptional regulation of MyoD1 and other muscle genes has not been addressed. Furthermore, it remains to be determined whether myogenic genes and the transcription factors SF1 and VDR are associated with protein–protein interaction complexes. Further studies should focus on understanding the molecular mechanism of the transcriptional regulation of MyoD1 and other muscle genes that interact with the transcription factors SF1 and VDR in skeletal muscle differentiation and development.

In conclusion, we successfully cloned the core region (nucleotides −420 to +17 bp) of the MyoD1 gene surrounding the transcription start site and found novel transcription factor binding sites, including VDR and SF1, in the Guanling cattle MyoD1 gene promoter by the promoter-binding TF profiling assay II. Myf6 strongly activated the MyoD1 promoter, while MyoD1 was also capable of efficiently activating the expression of its own promoter. The transcription factors MEF2A, SF1, and VDR were further confirmed to be capable of binding to MyoD1 by Y1H system experiments. Overexpression of the MyoD1 gene upregulated the expression of MEF2A, while it downregulated the mRNA expression of SF1 and VDR in the process of muscle myogenesis.

## 4. Experimental materials and methods

### 4.1 Experimental cattle and sampling

All procedures in the present study were approved by the Experimental Animal Management Committee of the Key Laboratory of Animal Genetics, Breeding, and Reproduction in the Plateau Mountainous Region, Guizhou University, Guiyang, China, and were performed according to the guidelines developed by the China Council of Animal Care.

Three 15 month old Guanling cattle (*N* = 3) were used for venous blood collection, according to the livestock slaughtering industry standards (GB/T 20551-2006) of the Chinese Ministry of Agriculture. Blood samples were first treated with heparin sodium anticoagulation and then stored at −70 °C for genomic DNA extraction using AxyPrepDNA Blood Genomic DNA Miniprep Kit (Axygen BioScience Inc, Union City, USA), according to the manufacturer's instructions. The three Guanling cattle were slaughtered following standard commercial procedures to collect the latissimus dorsi muscles. The fresh muscle tissues were placed in liquid nitrogen and stored at −80 °C for total RNA extraction.

### 4.2 Isolation of total RNA and genomic DNA as well as cDNA cloning

Total RNA was isolated from the latissimus dorsi samples using a TRIzol Regent Kit (Thermo Fisher Scientific, USA), according to the manufacturer's instructions. For cDNA cloning, the first-strand cDNA was synthesized from 1 μg of total RNA, according to the instructions of the Revert Aid™ First Strand cDNA Synthesis Kit (Thermo Fisher Scientific, USA).

Genomic DNA was extracted from the latissimus dorsi samples of Guanling cattle by using AxyPrep genomic DNA small kit (Item No. AP-MN-MS-GDNA-50; Axygen Biosciences, Union City, USA), according to the manufacturer's instructions. The quality of DNA was estimated by agarose gel electrophoresis.

### 4.3 PCR amplification of muscle-specific genes

The primers of bovine MyoD1 (NW_001040478), the MRF gene family (MyoD1: NM_001040478; Myf5: NM_174116; Myf6: NM_181811; MYOG: NM_001111325), the MEF2 gene family (MEF2A: NM_00108368; MEF2B: NM_001145793.1; MEF2C: NM_001046113.1; MEF2D: NM_001205178.1), the SF1 gene (NM_001081614.1), and the VDR gene (NM_001167932.1) were designed by Primer Premier 5.0 (http://www.premierbiosoft.com/primerdesign/index.html) and Oligo 6.0 (http://www.oligo.net/), according to the complete sequences from NCBI (Tables 1 and 2). The primers were synthesized by Shanghai Yingwei Jieji Biotechnology Co., Ltd. (Shanghai, China). PCR amplification was conducted using a 30 μL reaction mixture, including 15 μL of 2× Es Taq MasterMix, 3.0 μL of each of the reverse and forward primers (10 μM), 4 μL of template DNA (50 ng μL^−1^), and 8 μL of ddH_2_O. The reaction conditions were carried out with predenaturation at 94 °C for 2 min; followed by 35 cycles of 94 °C for 30 s, 58–62 °C (annealing temperature as shown in Tables 1 and 2) for 60 s, and 72 °C for 60 s; and finished at 72 °C for 5 min. The PCR products were stored at 4 °C. The restriction sites of the pGL3-MyoD1 promoter were kpnI and xhoI. The restriction sites of the vectors pcDNA3.1(+) – (MyoD1, Myf5, MEF2A, and SF1), pcDNA3.1(+) – (Myf6, MEF2B, and MEF2B), pcDNA3.1(+) – (MEF2C, VDR), and pcDNA3.1(+) – MyoG were EcoRI and xhoI, kpnI and EcoRI, BamHI and xhoI, and BamHI and EcoRI, respectively.

### 4.4 Screening of transcription factor binding sites in the bovine MyoD1 gene promoter

The transcription factor binding sites involved in regulating the bovine MyoD1 gene promoter were screened by using the promoter-binding TF profiling assay II (Catalog #: FA-2002; Signosis, Santa Clara, CA, USA) with the nuclear extracts from longissimus dorsi and purified target fragments recovered from PCR. In the screening, briefly, we used the promoter fragments and the fluorescently labelled probes to bind competitively to the protein in the nucleus extracts, and then the complex TF-bound probes were separated by denaturation using a set of procedures including column centrifugation and elution, according to the manufacturer's instructions (Signosis, Santa Clara, CA, USA). After denaturation, the complex compounds were incubated with a hybrid plate for measurement with a Multifunctional Microplate Reader (BioTek, Winooski, VT, USA). Androgen receptor (AR) was used as the blank control to normalize the readings that were statistically treated.

### 4.5 Statistical analysis

ABlank, ATreated, and AControl were the averages of the blank, control, and experimental groups, respectively. Whereas AControl was expressed as the readings of the control group minus ABlank, and ATreated was expressed as the readings of the experimental group minus AControl. The judgment value was calculated by AControl/ATreated. The statistically treated data were greater than 50, and the judgment value was greater than 3 in the present study.

### 4.6 Y1H assay

#### 4.6.1 Construction of bait vectors and prey plasmids

The PCR-amplified fragments of the MEF2A, SF1, and VDR were recovered from the gel, and ligated with the pGADT7 vector to construct the prey plasmids pGADT7-SF1, pGADT7-MEF2A, and pGADT7-VDR.

The PCR-amplified fragments of the MyoD1 gens and pAbAi were excised with the restriction enzyme, and then recovered by AxyPrep DNA Gel Recovery Kit (Item No. AP-GX-50; Axygen Biosciences, Union City, CA, USA), respectively. MyoD1 was ligated to pAbAi-1 to construct the bait vector pAbAi-MyoD1. The bait vector pAbAi-MyoD1 and the prey vector pGADT7-SF1 were transfected into the TOP10 strain and then spread transfected bacteria on lysogeny broth (LB) plates containing 1 mg mL^−1^ ampicillin for overnight inoculation at 37 °C. The individual colonies with a normal morphological size were picked for subculture in 20 mL of LB liquid medium overnight at 37 °C, from which the plasmids were extracted and verified by dual-enzyme digestion.

#### 4.6.2 Construction and identification of bait strains

The Y1H monoclone from yeast peptone dextrose adenine (YPDA) plates was inoculated in 15 mL of YPDA liquid medium at 30 °C with shaking at 200 rpm for 24 h, and 100 μL of culture was inoculated in 100 mL of YPDA solution at 30 °C with shaking at 200 rpm for 16–18 h. When the optical density at 600 nm (OD_600_) values were 0.8–1.0, the culture solution was centrifuged at 3000 rpm and 4 °C for 5 min. The sediment was resuspended in 50 mL of ice-cold sterile water, and centrifuged at 3000 rpm and 4 °C for 5 min. The step was repeated two more times. The last sediment was resuspended in 200 μL of ice-cold 1 M sorbitol and divided into two precooled Eppendorf® tubes containing 80 μL per tube. The prepared competent yeast cells were stored at −80 °C.

The pAbAi-1 plasmid was linearized with the Bbs1 enzyme, inoculated in a water bath overnight at 37 °C for recovery, and transfected into Y1H competent cells using an Eppendorf® electroporator with electroporation parameters of 1500 V and 5 min. A total of 150 μL of bacterial liquid was spread on the SD/-Ura plates and incubated at 30 °C for 4–5 days. After mixing well, the bacterial liquid was pipetted into 20 PCR tubes with 20 μL per tube that were placed in a PCR instrument for extension for 30 s together with a negative control and a positive control containing 0.5 μL of the corresponding plasmid, and assessed by agarose gel electrophoresis to identify positive transformants.

#### 4.6.3 Transformation of prey plasmids into bait strains

The positive monoclone on SD/-Ura plates (Section 4.6.2) was picked for culture in 15 mL of YPDA liquid medium at 30 °C with shaking at 200 rpm for 24 h, and 100 μL of culture was inoculated in 100 mL of YPDA solution at 30 °C with shaking at 200 rpm for 12–16 h. When the OD_600_ values were 0.8–1.0, the culture solution was centrifuged at 3000 rpm and 4 °C for 5 min. The supernatant was discarded, and the sediment was resuspended in 25 mL of ice-cold sterile water to centrifuge at 3000 rpm and 4 °C for 5 min. The step was repeated with two more times. The last sediment was resuspended in 200 μL of ice-cold 1 M sorbitol and divided into precooled 1 mL Eppendorf® tubes containing 80 μL per tube. The prepared competent yeast cells were stored at −80 °C.

The pGADT7 empty plasmid and pGADT7 prey plasmids were transfected into Y1H (pAbAi-1) competent cells using an Eppendorf® electroporator with electroporation parameters of 1500 V and 5 min, respectively. A 150 μL sample of the yeast cells was spread on SD/-Leu/AbA plates and incubated at 30 °C for 4–5 days. The bait strains were also spread on SD/-Leu/AbA plates as controls.

### 4.7 Analysis of double fluorescein in MyoD1 gene promoter-transfected cells

#### 4.7.1 Construction and identification of expression vectors and reporter plasmids

The PCR fragments containing the target promoter were sequenced and separated by 1.2% agarose gel electrophoresis, and the bands corresponding to the PCR products were excised from the gel. DNA samples were purified from the gel slices using an AxyPrep DNA Gel Recovery Kit, according to the manufacturer's instructions (Axygen Biosciences, Union City, CA, USA). The recovered target fragments were ligated to pcDNA3.1(+) and pGL3-basic using T4 DNA ligase overnight at 16 °C and then transformed into *E. coli*. The plasmid DNA samples were isolated from *E. coli* cultures, digested with restriction enzymes, and separated by electrophoresis on agarose gels to compare the fragment sizes to the target inserts. The plasmid DNA samples indeed harboring the target inserts were sequenced by Invitrogen to further confirm the identities of the target fragments, which were designated as pGL3-MyoD1 and pcDNA3.1(+) – SF1.

#### 4.7.2 Effect of the SF1 gene on MyoD1 gene promoter activity

The endotoxin-free plasmid DNAs were isolated from the plasmids pGL3-MyoD1, pcDNA3.1(+) – SF1, pcDNA3.1(+), and pGL3-basic as well as the internal reference vector pRL-TK by using an Endo-Free Plasmid Mini Kit I (Q-spin) (Omega, USA) (http://omegabiotek.com/store/product/plasmid-mini-kit-1-q-spin/), and the concentrations were estimated by a micro-spectrophotometer. Mouse C2C12 cells showing stable growth were inoculated onto 24-well cell culture plates at 1 × 10^5^ cells per well and incubated in an incubator at 37 °C, 5% CO_2_, and saturated humidity to 80% confluency for transfection with two groups of plasmid DNAs. The testing group consisted of 600 ng of pGL3-MyoD1 + 200 ng of pcDNA3.1(+) – SF1 + 60 ng of pRL-TK plasmid DNAs, while the control group contained 600 ng of pGL3-MyoD1 + 200 ng of pcDNA3.1(+) + 60 ng of pRL-TK plasmid DNAs. Each group was placed in three dual-wells and incubated in an incubator at 37 °C, 5% CO_2_, and saturated humidity for 24 h. The cell cultures were harvested for lysis with 120 μL of lysis buffer.

#### 4.7.3 Measurement of dual luciferase activity

Cell lysate (20 μL) was placed in a white 96-well microtiter plate to measure the luciferase activity using the Dual-Luciferase® Reporter Assay System (Promega, Madison, WI, USA), according to the manufacturer's instructions. Briefly, 100 μL of F assay reagent I was predispensed into the bottom of the tube, followed by gently tapping the tube wall for 3–5 times to mix, and put into a Multifunctional Microplate Reader (BioTek, Winooski, VT, USA) immediately to measure the luminous value (M1) in firefly luciferase luminous units (RLUs). A total of 100 μL of R assay reagent II was added the bottom of the tube, followed by gently tapping the tube wall for 3–5 times to mix, and put into a multifunctional microplate reader immediately to record the luminous value (M2) in RLUs. The relative luciferase activity, calculated by M1/M2, was presented as the mean ± standard deviation of three replicates.

### 4.8 Overexpression of the MyoD1 gene regions

The cells in a 10 cm Petri dish were grown to about 80% confluency and digested by 0.25% trypsin for cell counting. Then, the cells were transferred to a 24-well plate at 5 × 10^4^ cells per well and inoculated overnight in 10% fetal bovine serum (FBS) complete medium. Puromycin was prepared as a 1 mg mL^−1^ stock solution and diluted with FBS complete medium, followed by addition to wells at working concentrations of 0, 0.2, 0.4, 0.8, 1, 1.5, 3, and 10 μg mL^−1^. The plate was inoculated in an incubator to observe cell growth over 3 days for screening of the lowest concentration that kills all cells. In the present study, 1 μg mL^−1^ puromycin was used as the screening concentration.

The target cells were transferred to a 24-well plate at 5 × 10^5^ cells per well and cultured overnight in 10% FBS medium. Meanwhile, the lentivirus solution was removed from −80 °C and thawed in an ice-water bath. Once the virus was fully thawed, the proper amount was transferred to a well of a 24-well plate and diluted with FBS medium to 1 × 10^7^ virus per well to bring up the volume in the wells to 1 mL containing 2 μg mL^−1^ polybrene. Six hours after viral infection, the medium was removed and replaced with 2 mL of FBS complete medium, and the cells were incubated in an incubator. One well was used as uninfected control cells. At 48 h after infection, the medium was replaced with screening medium containing 1 μg mL^−1^ puromycin, and the cells were cultured in an incubator and observed daily. The screening medium was replaced daily for 3–5 days when the control cells were completely dead. The infected cells were continuously cultured on maintenance medium containing 0.5 μg mL^−1^ puromycin to grow to the desired density. Two days later, the infected cells in each well of a 6-well plate were transferred to two wells, and the cells were continuously cultured on maintenance medium for 2–3 days. Then, the cells from one well were examined, and those from another well continued to be cultured. Ten days after antibiotic screening, the cells were collected for detection of the expression of the target genes using Q-PCR SsoFast™ EvaGreen Supermix (Item No: 172-5201AP; BIO-RAD, Hercules, CA, USA). Stable cell lines were frozen for future use.

## Author contributions

Houqiang Xu and Wei Chen conceived and designed the experiments; Di Zhou, Tao Yang, Ming Zhang performed the experiments; Di Zhou and Yuanyuan Wang analyzed the data; Di Zhou wrote the paper.

## Conflicts of interest

The authors declare that no conflicting interests exist.

## Supplementary Material
