# Correction: Study on the transcriptional regulatory mechanism of the MyoD1 gene in Guanling bovine

**DOI:** 10.1039/c8ra90096e

**Published:** 2018-12-13

**Authors:** Di Zhou, Houqiang Xu, Wei Chen, Yuanyuan Wang, Ming Zhang, Tao Yang

**Affiliations:** Key Laboratory of Animal Genetics, Breeding and Reproduction in the Plateau Mountainous Region, Cell and Molecular Biology (PhD), Animal Department, Guizhou University Guiyang 550025 China dizhougz@163.com; College of Life Science, Guizhou University Guiyang 550025 China

## Abstract

Correction for ‘Study on the transcriptional regulatory mechanism of the MyoD1 gene in Guanling bovine’ by Di Zhou *et al.*, *RSC Adv.*, 2018, **8**, 12409–12419.

The authors regret that incorrect details were given for ref. 56 in the original article. The correct version of ref. 56 is given below.

The Royal Society of Chemistry apologises for these errors and any consequent inconvenience to authors and readers.

## Supplementary Material
